# Outcomes in Patients Receiving Treatment for Pulmonary Arterial Hypertension Associated With Repaired Congenital Heart Disease

**DOI:** 10.1016/j.jacadv.2025.101626

**Published:** 2025-02-24

**Authors:** Richard A. Krasuski, Tobore Tobore, Sean Studer, Pavel Jansa, Olivier Sitbon, Marius M. Hoeper, Richard Channick, Sean Gaine, Irene Lang, Kelly Chin, Tomas Pulido, Sanjay Mehta, Adam Torbicki, Bhagavatula Sastry, Xiaoqin Tang, Vallerie McLaughlin, Leigh C. Reardon

**Affiliations:** a Duke University Health System, Durham, North Carolina, USA; b Johnson & Johnson, Titusville, New Jersey, USA; c Charles University and General University Hospital, Prague, Czech Republic; d Université Paris-Saclay, APHP, Hôpital Bicêtre, Le Kremlin-Bicêtre, France; e Hannover Medical School, Hannover, Germany; f German Center of Lung Research (DZL), Hannover, Germany; g David Geffen School of Medicine at University of California Los Angeles, Los Angeles, California, USA; h Mater Misericordiae University Hospital, Dublin, Ireland; i Cardiology and Center of Cardiovascular Medicine, Medical University of Vienna, Vienna, Austria; j University of Texas, Southwestern Medical Center, Dallas, Texas, USA; k Ignacio Chavez National Heart Institute, Mexico, Mexico; l London Health Sciences Centre, Schulich School of Medicine and Dentistry, Western University, London, Ontario, Canada; m Centre of Postgraduate Medical Education, European Health Centre, Otwock, Poland; n CARE Hospitals, Hyderabad, India; o University of Michigan Health System, Ann Arbor, Michigan, USA; p Ahmanson/UCLA Adult Congenital Heart Disease Center University of California, Los Angeles, California, USA

**Keywords:** COMPASS-2, congenital heart disease, GRIPHON, pooled analysis, pulmonary arterial hypertension, SERAPHIN

## Abstract

**Background:**

Pulmonary arterial hypertension (PAH) is a common complication among patients with congenital heart disease (CHD). Despite advances in PAH treatment, evidence for the benefits of PAH therapies in CHD-PAH is limited.

**Objectives:**

This analysis aimed to evaluate outcomes in patients with repaired PAH-CHD receiving an approved PAH drug.

**Methods:**

This was a pooled analysis including CHD-PAH patients whose CHD was repaired ≥1 year prior from 3 randomized, placebo-controlled, event-driven studies: GRIPHON (NCT01106014), SERAPHIN (NCT00660179), and COMPASS-2 (NCT00303459). The primary endpoint was time to first confirmed morbidity/mortality (M/M) event. HRs with 95% CIs were determined with random effects models.

**Results:**

The analysis included 1,982 patients with PAH, 177 (8.9%) with CHD-PAH. In the overall PAH cohort, the mean age was 48 and 49 years in treatment and placebo groups; 80% and 77% were female. In the CHD-PAH cohort, the mean age was 41 and 39 years; 70% and 66% were female. Overall, ≥98% in each group were World Health Organization functional class II and III at baseline. There was a significant reduction in risk of M/M events vs placebo in the overall PAH and CHD-PAH cohorts: 37% reduction in the overall PAH cohort (HR: 0.63; 95% CI: 0.52-0.77) and 50% reduction in the CHD-PAH population (HR: 0.50; 95% CI: 0.26-0.94).

**Conclusions:**

Treatment with approved PAH drugs provided a similar reduction in M/M risk in patients with repaired CHD-PAH when compared with the overall PAH population. This pooled analysis provides important evidence to guide medical management in this patient population.

Patients with congenital heart disease (CHD) are at risk of developing pulmonary arterial hypertension (PAH), which occurs in around 3% to 7% of adults with CHD and increases their all-cause mortality rate almost 2-fold.^1^, ^2^, ^3^, ^4^ Real-world and registry data from Europe and North America indicate that 5% to 10% of all PAH patients have CHD-associated PAH (CHD-PAH),^5^, ^6^, ^7^ and a Chinese registry suggested that CHD-PAH was the most common PAH etiology in China (45% of patients).^8^ The population of patients with CHD has increased over recent decades due to improvements in diagnosis and survival resulting from advances in cardiac and surgical care,^9^ and the prevalence of CHD-PAH is also increasing.^10^ Patients with CHD-PAH appear to have longer survival than patients with idiopathic and other PAH etiologies.^6^^,^^11^^,^^12^

CHD-PAH is a heterogeneous condition encompassing patients with Eisenmenger syndrome (an advanced form of CHD-PAH), unrepaired systemic-to-pulmonary shunts, small/coincidental defects, or PAH persisting or recurring after defect repair.^2^ Patients who develop CHD-PAH after surgical repair may have a poorer prognosis compared with other CHD-PAH subgroups.^12^^,^^13^ Therefore, there is a need for early and effective detection and management of patients with repaired defects and CHD-PAH.^2^

New medical therapies have improved the outcomes of patients with PAH.^2^^,^^14^, ^15^, ^16^ However, there are limited data from randomized controlled trials (RCTs) on outcomes in adults with CHD-PAH for the currently approved PAH treatments. Most of the evidence supporting the use of medical PAH therapies in adults with repaired defects and CHD-PAH comes from subgroup analyses of RCTs conducted in a wider population of patients with PAH.^14^, ^15^, ^16^, ^17^ A small percentage of patients with repaired CHD were included in these studies. The available evidence supported benefits in the subgroup of patients with repaired CHD who received an active PAH drug in COMPASS-2 (Cardiovascular Outcomes for People Using Anticoagulation Strategies) (20 patients with repaired CHD-PAH),^14^ SERAPHIN (Study with an Endothelin Receptor Antagonist in Pulmonary Arterial Hypertension to Improve Clinical Outcome) (47 patients with repaired CHD-PAH),^15^ and GRIPHON (Prostacyclin [PGI2] Receptor Agonist in Pulmonary Arterial Hypertension) (110 patients with repaired CHD-PAH).^16^^,^^17^

To estimate the treatment benefits of PAH therapy more accurately in adult patients with repaired CHD-PAH, we conducted a pooled analysis of individual patient data for patients with repaired CHD-PAH enrolled in SERAPHIN, GRIPHON, and COMPASS-2. The treatment benefit was assessed by analysis of the risk of a first mortality/PAH-related morbidity event (primary endpoint), functional outcomes (6-minute walk distance [6MWD], Borg dyspnea index, World Health Organization functional class [WHO FC], *N*-terminal prohormone of brain natriuretic peptide [NT-proBNP]), and pharmacoeconomic parameters (PAH-related hospitalizations).

## Methods

### Study design

This pooled analysis included individual data from patients enrolled in 3 randomized, placebo-controlled studies evaluating currently approved PAH treatments: GRIPHON (NCT01106014),^16^ SERAPHIN (NCT00660179),^15^ and COMPASS-2 (NCT00303459)^14^ in patients with PAH. These studies were selected for inclusion in the current pooled analysis because of the similarities in study design (all were event-driven trials with a similar primary endpoint) and timeframes of study conduct (Table 1), as well as the availability of individual patient data in the clinical database for each study (allowing data pooling by the sponsor). The overall objective of the pooled analysis was to evaluate the benefits of PAH-specific drugs in patients with CHD-PAH. Pooled data were used to provide a cohort of patients with CHD-PAH that was sufficiently sized to evaluate treatment benefits among this small patient subgroup enrolled in each study (∼10% of patients in GRIPHON, 8% in SERAPHIN, and 6% in COMPASS).^14^, ^15^, ^16^ Pooled data for the overall PAH population were also analyzed to provide a benchmark for assessing the results in the CHD-PAH cohort. Each study protocol was approved by the relevant Institutional Review Board and conducted in accordance with the ethical principles of the Declaration of Helsinki, Good Clinical Practices, regulatory requirements, and International Society for Heart and Lung Transplantation ethics. All patients provided written informed consent before participation in the clinical trials used in this analysis.

**Table 1:** Study Patient Populations Included in the Pooled Analysis

Study, Recruitment Time Frame, and Active Drug	CHD-PAH Patient Subpopulation in Each Trial	Morbidity/Mortality Event Definition for Primary Endpoint	Overall PAH Cohort (n = 1,982)		CHD-PAH Cohort (n = 177)	
			Active Drug	Placebo	Active Drug	Placebo
GRIPHON ^16^ December 2009 to May 2013 Selexipag	CHD-PAH with repaired congenital systemic-to-pulmonary shunts (≥1 year repaired)	• Death • Hospitalization for worsening of PAH • Need for lung transplantation or balloon atrial septostomy • Initiation of prostanoids or chronic O_2_ therapy • Disease progression (6MWD/WHO FC/add PAH therapy)	574 (29.0)	582 (29.4)	60 (33.9)	50 (28.2)
SERAPHIN^a^^15^ May 2008 to December 2009 Macitentan	CHD-PAH with repaired congenital systemic-to-pulmonary shunts (≥1 year repaired)	• Death • Atrial septostomy or lung transplantation (or hospitalization for such events) • Initiation of IV/SC prostanoids • Worsening of PAH (6MWD + WHO FC or signs and symptoms of RHF not responding to optimal diuretics + need for new PAH therapy)	242 (12.2)	250 (12.6)	21 (11.9)	26 (14.7)
COMPASS-2 ^14^ May 2006 to June 2012 Bosentan	CHD-PAH with repaired congenital heart defect (≥2 years repaired)	• Death • Hospitalization for worsening PAH or start of IV prostanoid, atrial septostomy, lung transplant, or worsening PAH (defined as: worsening of PAH [patient assessment] or 6MWD + SC/inhaled prostanoid or use of bosentan)	159 (8.0)	175 (8.8)	9 (5.1)	11 (6.2)

Values are n (%).

6MWD = 6-minute walk distance; CHD = congenital heart disease; FC = functional class; IV = intravenous; PAH = pulmonary arterial hypertension; RHF = right heart failure; SC = subcutaneous; WHO = World Health Organization.

a Only patients receiving macitentan at the 10-mg dose were included in the active treatment group.

### Patients

The inclusion/exclusion criteria for each study included in the pooled analysis have been described in detail previously.^14^, ^15^, ^16^ In all 3 studies, eligible patients (aged 18-75 years in GRIPHON, ≥12 years in SERAPHIN, and ≥18 years in COMPASS-2) had idiopathic or heritable PAH or PAH associated with HIV, drug use or toxin exposure, connective tissue disease, or repaired congenital systemic-to-pulmonary shunts. Patients with CHD were required to be ≥ 1 year out from repair of their congenital shunt prior to study enrollment (and ≥2 years in COMPASS-2).^14^ Confirmation of PAH by right heart catheterization and a 6MWD of ≥50 m (150 m in COMPASS-2) were also required. Concomitant treatment with a stable dose for ≥3 months of the following PAH therapies was permitted: endothelin receptor antagonist and/or phosphodiesterase five inhibitor in GRIPHON, any oral or inhaled PAH therapy other than an endothelin receptor antagonist in SERAPHIN, and sildenafil in COMPASS-2.

### Endpoints and outcome measures

The primary endpoint of the analysis was the time to the first mortality and morbidity (M/M) event by the end of treatment (+7 days), confirmed by clinical event committee. Morbidity events were disease progression or PAH worsening leading to hospitalization, parenteral prostanoid initiation, or long-term oxygen therapy, and the need for lung transplantation or atrial septostomy (per the definition used in each study; see Table 1). The end of the treatment period occurred at the completion of the study (for patients who did not have a primary endpoint event), after the occurrence of a primary endpoint event, or prematurely for different reasons, including an adverse event.

Predefined secondary endpoints were the composite endpoint of time to PAH-related death or hospitalization for PAH worsening (a component of the M/M primary endpoint), as well as individual endpoints assessing changes in 6MWD, Borg dyspnea score, WHO FC, and NT-proBNP at month 6 vs at the initiation of study treatment. Pharmacoeconomic endpoints were the annualized number of PAH-related hospitalizations and number of days spent in the hospital for PAH-related causes up to the end of study treatment.

### Statistical analyses

An individual patient data pooled analysis was conducted by synthesizing raw individual-level patient data from the 3 studies using a 1-stage framework. Individual patient data were analyzed simultaneously by within-study clustering of participants using generalized linear (mixed) models with random effects to allow for variation across studies, methodology previously validated for 1-stage individual patient data meta-analysis models with maximum likelihood estimation.^18^^,^^19^

All parameters and endpoints were analyzed in the CHD-PAH and overall PAH cohorts. Covariates for adjusted analyses comprised the following demographic and baseline characteristics (and to explore their relationship to outcomes): age, sex, race (White, non-White), time since diagnosis of PAH, WHO FC at the time of study treatment initiation (regrouped as WHO FC I/II and III/IV), 6MWD, Borg dyspnea index, log-transformed NT-proBNP, and concomitant therapies.

All analyses were carried out according to the intention-to-treat principle. For time-to-event endpoints, effects of treatment were quantified by estimating HRs using mixed effects Cox models. Continuous outcomes were quantified by least-squares mean difference using linear mixed effects models. Binary outcomes were analyzed with generalized linear mixed models with estimated ORs.

Changes in 6MWD and Borg dyspnea index were calculated as the difference between measurement at month 6 vs at the initiation of study treatment. WHO FC worsening or improvement was defined as any decrease (FC improvement) or increase (FC worsening) in FC at month 6 vs a patient’s FC at the initiation of study treatment. Patients with missing WHO FC at month 6 were treated as a worsening event. Change in NT-proBNP from baseline to month 6 was calculated as the ratio to baseline value.

Rates of hospitalization and numbers of hospital days (PAH-related hospitalizations) up to end of treatment were assessed descriptively in terms of patient-years of exposure in each treatment arm and analyzed using generalized linear mixed regression models for count data (a Poisson regression model for the rates of hospitalization and a hurdle negative binomial model for the number of hospital days, as used previously).^20^ Treatment effects on the rates of hospitalization and number of hospital days were expressed as rate reductions with 95% CI.

All analyses were performed in R for Windows, version 4.2.1 (R Development Core Team 2022. R Foundation for Statistical Computing).

## Results

### Analysis cohorts

In total, 1,982 patients were included in the overall PAH cohort, including 177 (8.9% of the overall cohort) with repaired CHD-PAH (Table 1). In the overall PAH cohort, 975 (49.2%) patients received active drug and 1,007 (50.8%) received placebo, with similar proportions in the CHD-PAH cohort (90 [50.8%] active drug; 87 [49.2%] placebo). A similar proportion of CHD patients and ratio of treatment compared with placebo were included from each study.

The most common repaired CHD subtypes in the individual studies among patients with CHD-PAH were atrial septal defect (43.6%, 42.6%, and 70.0% in GRIPHON, SERAPHIN, and COMPASS-2, respectively), ventricular septal defect (10.9%, 19.1%, and 5.0%), and patent ductus arteriosus (7.3%, 6.4%, and 0%).

### Baseline patient characteristics

Patient demographics and baseline characteristics were well balanced between active drug and placebo treatment arms in both the overall PAH and the CHD-PAH cohorts (Table 2). The mean age of patients at study enrollment was lower in the CHD-PAH cohort; for those patients receiving active drug, the mean age was 41 years in the CHD-PAH cohort vs 48 years in the overall PAH cohort. In both the overall PAH and CHD-PAH cohorts, most patients were female (78% and 77%, respectively), White (66% and 61%), and in WHO FC II/III (98% and 99%). Baseline characteristics in the overall PAH and CHD-PAH cohorts displayed according to the study in which patients were enrolled are provided in Supplemental Tables 1 and 2. There were no notable differences in the characteristics of the patients included in the overall PAH cohort from the 3 randomized studies. In the CHD-PAH cohort, demographic and disease characteristics were generally similar between PAH-specific drug and placebo groups across the 3 studies; however, in SERAPHIN, the placebo group had a younger age, higher proportion of males, and longer time from diagnosis to treatment initiation than the active PAH drug group (Supplemental Table 2).

**Table 2:** Patient Demographics and Baseline Clinical Characteristics in the Pooled Analysis Cohorts

	Overall PAH Cohort		Repaired CHD-PAH Cohort	
	Active Drug (n = 975)	Placebo (n = 1,007)	Active Drug (n = 90)	Placebo (n = 87)
Age, y	48 ± 15	49 ± 16	41 ± 16	39 ± 15
Female	776 (80)	779 (77)	70 (78)	66 (76)
Race				
White	658 (67)	655 (65)	55 (61)	53 (61)
Non-White	317 (33)	352 (35)	35 (39)	34 (39)
BMI, kg/m^2^	27 (6)	27 (6)	26 (6)	24 (5)
Time from PAH diagnosis, months	28 (43)	29 (46)	49 (81)	56 (82)
WHO FC				
I	5 (<1)	5 (<1)	1 (1)	0 (0)
II	465 (48)	454 (45)	54 (60)	50 (57)
III	497 (51)	534 (53)	35 (39)	37 (43)
IV	8 (<1)	14 (1)	0	0
Concomitant PAH therapy				
Missing	5 (<1)	0	0	0
No	195 (20)	220 (22)	29 (32)	28 (32)
Yes	775 (79)	787 (78.2)	61 (68)	59 (68)
6MWD				
Mean ± SD	362.3 ± 79.5	354.0 ± 87.4	379.9 ± 72.9	376.0 ± 83.3
Median (Q1, Q3)	379 (315,421)	370 (300,420)	387.5 (346,435.8)	387 (335,435)

Values are n (%), mean ± SD, or median (Q1, Q3).

BMI = body mass index; CHD = congenital heart disease; other abbreviations as in Table 1.

Subtypes of CHD included in the study were very heterogeneous. Congenital diseases present in the overall study populations are summarized in Supplemental Table 3 (GRIPHON), Supplemental Table 4 (SERAPHIN), and Supplemental Table 5 (COMPASS-2).

### Risk of morbidity/mortality events (primary endpoint)

**Figure 1:**
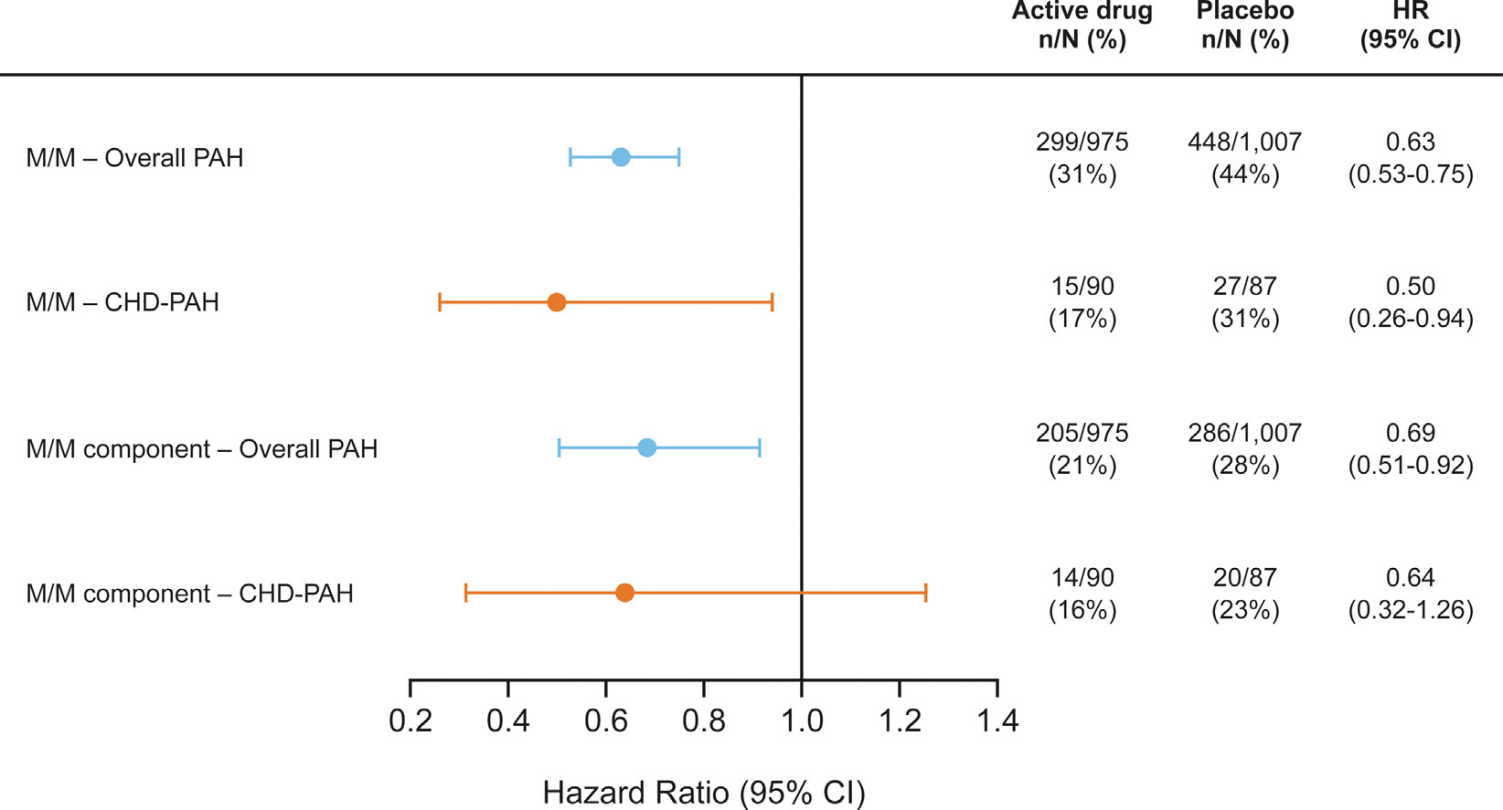
**Risk of Morbidity/Mortality Events in the Overall Pulmonary Arterial Hypertension and Repaired Congenital Heart Disease-Pulmonary Arterial Hypertension Cohorts** HRs are based on a mixed effect Cox model, adjusted for age, sex, and time from diagnosis. M/M events are described in Table 1. Analysis was a composite of 2 components of the M/M endpoint, including death due to PAH or hospitalization due to PAH worsening. CHD = congenital heart disease; M/M = morbidity/mortality; n = number of patients with events; N = number of patients at risk; PAH = pulmonary arterial hypertension.

In the overall PAH cohort, 299 (31%) patients receiving active drug experienced an M/M event vs 448 (44%) patients receiving placebo. Corresponding values were 15 (17%) and 27 (31%) in the CHD-PAH cohort. Analysis of M/M risk using a mixed effects Cox model (adjusted for age, sex, and time from diagnosis) showed that treatment with active drug vs placebo provides a risk reduction in the CHD-PAH cohort (HR: 0.50; 95% CI: 0.26-0.94), similar to that in the overall PAH cohort (HR: 0.63; 95% CI: 0.53-0.75) (Figure 1).

A similar analysis of a composite of 2 components of the M/M endpoint—death due to PAH or hospitalization due to PAH worsening (secondary endpoint)—also showed that risk reduction with active drug vs placebo was similar in the CHD-PAH (HR: 0.64; 95% CI: 0.32-1.26) and overall PAH cohorts (HR: 0.69; 95% CI: 0.51-0.92) (Figure 1).

### Secondary clinical endpoints: 6MWD, NT-proBNP, WHO FC

In the overall PAH cohort, the difference between the active drug and placebo groups in mean change in 6MWD from baseline to month 6 was 11.9 m (95% CI: −1.5 to 26.4) in the mixed effects model (adjusted for baseline 6MWD, age, sex) (Table 3), and 13.4 m (95% CI: 2.1-24.7) in the sensitivity analysis using the stratified model (including fixed study-specific intercepts and random treatment effects). A similar benefit was seen in the CHD-PAH cohort, with a mean difference in the change from baseline at month 6 between active drug and placebo of 20.1 m (95% CI: −15.9 to 56.2) in the random intercept-only model. Due to the small sample size of the CHD-PAH cohort, mixed and stratified models did not converge.

**Table 3:** Secondary Endpoints in the Overall PAH and Repaired CHD-PAH Cohorts

	Overall PAH Cohort			Repaired CHD-PAH Cohort		
	Active (n = 975)	Placebo (n = 1,007)	Difference	Active (n = 90)	Placebo (n = 87)	Difference
	Mean ± SD	Mean ± SD	LSMD (95% CI)	Mean ± SD	Mean ± SD	LSMD (95% CI)
Change in 6MWD	−25.0 ± 129.5	−38.6 ± 128.7	11.9 (1.5 to 26.4)	−3.9 ± 112.4	−18.6 ± 130.5	20.1 (−15.9 to 56.2)
Change in NT-proBNP, ratio of baseline	1.21 ± 2.50	1.36 ± 1.14	0.76 (0.71-0.81)	1.14 ± 0.75	1.18 ± 0.62	0.92 (0.77-1.10)
Change in Borg dyspnea score	−0.22 ± 1.68	0.09 ± 1.69	−0.28 (−0.43 to −0.13)	−0.54 ± 1.59	−0.15 ± 1.37	−0.22 (−0.63 to 0.19)

	Rate ± SD	Rate ± SD	IRR (95% CI)	Rate ± SD	Rate ± SD	IRR (95% CI)
Annual rate of PAH hospitalizations	0.39 ± 1.76	0.60 ± 2.08	0.81 (0.56-1.17)	0.16 ± 0.48	0.87 ± 4.47	0.55 (0.29-1.03)
Annualized PAH-related hospital days	15.5 ± 28.2	20.1 ± 34.6	0.93 (0.51-1.71)	8.9 ± 10.5	29.2 ± 62.5	0.23 (0.04-1.25)

CHD = congenital heart disease; IRR = incidence rate ratio; LSMD = least-square mean difference; NT-proBNP = *N*-terminal prohormone of brain natriuretic peptide; other abbreviations as in Table 1.

Table 3 shows results for changes in NT-proBNP analyzed as the ratio of active drug and placebo of the geometric mean values for NT-proBNP at month 6 (calculated as the ratio to baseline). In the overall PAH cohort, the ratio was 0.76 (95% CI: 0.71-0.81) in the mixed effects model (adjusted for age, sex), with identical results seen in the sensitivity analysis using the stratified model. In the CHD-PAH group, the ratio was 0.92 (95% CI: 0.77-1.10) in the random intercept model, with similar findings in the stratified model. Small treatment differences were seen with the mean change in Borg dyspnea index from baseline to month 6, with a similar difference (improvement) between active drug and placebo evident in the overall PAH cohort (−0.28; 95% CI: −0.43 to −0.13) and the CHD-PAH cohort (−0.22; 95% CI: −0.63 to 0.19) for the random intercept model adjusted for age and baseline Borg dyspnea index.

The OR for WHO FC worsening (active drug vs placebo) was 0.84 (95% CI: 0.53-1.33) in the mixed model (adjusted for age, sex, baseline WHO FC III/IV) in the overall PAH group and 0.99 (95% CI: 0.41-2.37) in the CHD-PAH cohort, indicating no difference (Table 4).

**Table 4:** Analyses of WHO FC Worsening in the Pooled Analysis Cohorts

	Overall PAH Cohort		Repaired CHD-PAH Cohort	
	Active Drug	Placebo	Active Drug	Placebo
WHO FC worsening from baseline to month 6 (with all patients discontinuing prior to month 6 analyzed as a worsening event)	n = 975	n = 1,007	n = 90	n = 87
Patients with nonmissing values	829 (85)	824 (82)	83 (92)	75 (86)
Patients with worsening	196 (20)	243 (24)	12 (13)	12 (14)
Patients without worsening	779 (80)	764 (76)	78 (87)	75 (86)
Risk of WHO FC worsening (active treatment vs placebo)^a^	0.84 (0.53-1.33)		0.99 (0.41-2.37)	

Values are n (%) or OR (95% CI).

CHD = congenital heart disease; other abbreviations as in Table 1.

a Mixed model adjusted for age, sex, and baseline WHO FC 3/4.

### Pharmacoeconomic endpoints

A similar treatment effect for the number of PAH-related hospitalizations and PAH-related hospital days was seen in the overall PAH and the CHD-PAH cohorts (Table 3). PAH-related hospitalizations occurred in 200 (20.5%) and 264 (26.2%) patients in the active drug and placebo groups in the overall PAH cohort; and in 16 (17.8%) and 20 (23%), respectively, in the CHD-PAH cohort. The annualized incident rate ratio (IRR; active drug vs placebo) for hospitalizations using a mixed effect model was 0.81 (95% CI: 0.56-1.17) in the overall PAH cohort and 0.55 (95% CI: 0.29-1.03) in the CHD-PAH cohort.

Most patients were not hospitalized. A hurdle negative binomial model, in the overall PAH group, showed that patients in the active treatment group were less likely to be hospitalized (active drug vs placebo IRR 1.39, 95% CI: 1.12-1.71; note, that this IRR indicates the risk of no hospitalization). A similar analysis in the smaller CHD-PAH group showed no treatment difference (active drug vs placebo, IRR 1.38; 95% CI: 0.66-2.88). Once a patient was hospitalized, there was no evidence of treatment benefit in terms of days spent in hospital: the active drug vs placebo IRR for the annualized number of PAH-related hospital days was 0.93 (95% CI: 0.51-1.71) in the overall PAH group and 0.23 (95% CI: 0.04-1.25) in the CHD-PAH cohort (Table 3). Among hospitalized patients, the mean annualized number of PAH-related hospital days with active drug vs placebo was 15.5 ± 28.2 days vs 20.1 ± 34.6 days in the overall PAH group and 8.9 ± 10.5 days vs 29.2 ± 62.5 days in the CHD-PAH cohort.

## Discussion

This pooled analysis using data from 3 randomized trials confirms that treatment with PAH-approved drugs reduces the M/M risk specifically in patients with repaired CHD-PAH, with the magnitude of treatment benefit similar to that observed in the overall PAH population (Central Illustration). Comparison of the group receiving PAH treatment vs placebo showed a 50% reduction in the risk of an M/M event in the CHD-PAH cohort (HR: 0.50; 95% CI: 0.26-0.94) and a 37% reduction in the overall PAH cohort (HR: 0.63; 95% CI: 0.52-0.77).

**Central Illustration:**
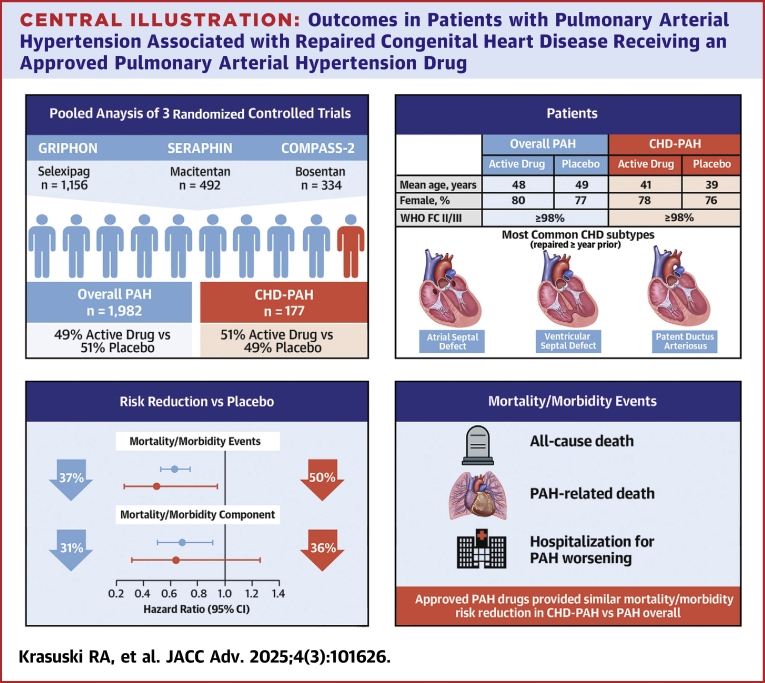
**Outcomes in Patients with Pulmonary Arterial Hypertension Associated with Repaired Congenital Heart Disease Receiving an Approved Pulmonary Arterial Hypertension Drug** FC = functional class; WHO = World Health Organization; other abbreviations as in Figure 1.

The more robust point estimate for M/M risk reduction in the pooled analysis augments the evidence derived from subgroup analyses in individual RCTs. For example, in SERAPHIN, the point estimate for M/M risk reduction with active drug vs placebo in the CHD-PAH subgroup (HR: 0.58; 95% CI: 0.25-1.37) was in line with the 45% risk reduction seen in the overall population (HR: 0.55; 97.5% CI: 0.39-0.76; *P* < 0.001).^15^ However, the point estimate in the CHD-PAH subgroup was based on a very small number of events (10 events in 26 patients on placebo, four events in 21 patients on active PAH drug) and the point estimate for M/M risk reduction had very wide confidence intervals (with an upper limit that crossed zero), so it was not possible to definitively conclude that efficacy was similar in the CHD-PAH subgroup and the overall PAH population. Our analysis provides robust point estimates, with narrow confidence intervals, indicating that PAH treatment reduces the risk of an M/M event in both the CHD-PAH and overall PAH cohorts and provides important evidence to guide medical management of patients with CHD-PAH.

Our analyses of secondary endpoints confirmed that PAH treatment provides statistically significant benefits vs placebo on functional parameters (6MWD, Borg dyspnea index) and cardiac biomarkers (NT-proBNP) in the overall PAH cohort. Similar analyses in the CHD-PAH cohort did not show statistically significant treatment differences, but the magnitude of the treatment effect was generally similar to that seen in the overall cohort. The secondary endpoint analyses in the CHD-PAH cohort were limited by the relatively small sample size, and this was particularly evident in the analysis of WHO FC worsening (with events in only five patients on active drug and no patients on placebo in the CHD-PAH cohort, when patients with missing data were excluded from the analysis). Active PAH drug treatment showed benefits in terms of reducing the rate of PAH-related hospitalizations vs placebo in both the overall PAH and CHD-PAH cohorts. However, this observation should be interpreted with caution, as fewer than a quarter of patients had a PAH-related hospitalization in the overall PAH and CHD-PAH cohorts, and hospital stay is likely country-specific, with some countries tending to keep patients longer in hospital even if the condition is not severe.

The patient population in our CHD-PAH pooled analysis cohort is representative of the enrollment criteria of the 3 contributing RCTs. In GRIPHON and SERAPHIN, CHD-PAH patients were required to have undergone CHD repair ≥1 year prior to enrollment, with patients in COMPASS-2 required to have the defect repaired ≥2 years prior.

Our study used a well-validated individual patient data pooled analysis method, involving the synthesis of raw individual-level patient data from the 3 studies using a 1-stage approach accounted for between study heterogeneity with a mixed effects model.^18^^,^^19^ The 3 RCTs providing data enrolled similar patient populations, had a similar randomized, event-driven study design, and a similar M/M primary endpoint.

### Study limitations

Our study has several limitations. Selection of studies for the pooled analysis was based on access to individual patient data from the sponsor, which allowed pooling of similar data, and could have introduced potential for bias. We also acknowledge that enrolled patients were potentially selected for survival after repair of CHD. Another limitation is that, despite the pooling of 3 RCTs, the small size of the CHD-PAH cohort may have compromised the analysis of the secondary endpoints, and the effect size is small in absolute numbers and insufficient to inform routine clinical practice. Given the different nature of the active drugs evaluated in the 3 RCTs, a pooled analysis of safety was not feasible. Furthermore, genetic data were not available for all patients and therefore could not be analyzed in this study.

## Conclusions

Treatment with PAH-approved drugs provided a similar reduction in M/M risk in patients with repaired CHD-PAH when compared with the overall PAH population. This pooled analysis using data from 3 randomized trials indicates that there is an improvement in outcomes with PAH therapy in patients with CHD-PAH and provides important evidence to guide medical management of this growing patient population.

Perspectives **COMPETENCY IN MEDICAL KNOWLEDGE:** Patients with repaired CHD-PAH derive similar benefit from PAH medical treatments as the overall population of patients with PAH.

**TRANSLATIONAL OUTLOOK:** Further research is needed to understand whether improvements in clinical and functional parameters accompany the significant reduction in the risk of M/M achieved with PAH treatment in patients with CHD-PAH.

## Funding support and author disclosures

This study was funded by Johnson & Johnson. Drs Tobore, Studer, and Tang are employees of the funding organization and as study contributors were involved in the collection of data, its analysis and interpretation, and had the right to approve or disapprove publication of the finished manuscript. Dr Krasuski has served as a consultant for Johnson & Johnson, Bayer, Gore Medical, and Neptune Medical; has received research grants from Johnson & Johnson and the Adult Congenital Heart Association; and has served as an investigator in studies by Artivion, Corvia, Medtronic, and Edwards Lifesciences. Dr Jansa has received fees and grants from Johnson & Johnson, AOP Orphan, Bayer Healthcare, MSD, and Arena Pharmaceuticals Inc. Dr Sitbon has received fees for serving on a steering committee from Janssen Johnson & Johnson (formerly Actelion) and Gossamer Bio; fees for writing assistance from Johnson & Johnson (formerly Actelion); consulting and lecture fees from Johnson & Johnson (formerly Actelion), AOP Orphan, Ferrer, and MSD; and fees for serving on advisory boards from Actelion, AOP Orphan, Enzyvant, Ferrer, Gossamer Bio, Liquidia, MSD, Respira Therapeutics, Roivant, and United Therapeutics. Dr Hoeper has received fees for lectures or consultations from Acceleron, Actelion, AOP, Bayer, Ferrer, Gossamer, Johnson & Johnson, Keros, and MSD. Dr Channick has served as a steering committee member for Johnson & Johnson; has served on an advisory board for Johnson & Johnson, Merck, and Bayer; has received consultancy fees from Bayer, Gossamer, Merck, and Arena Pharmaceuticals; has received support for attending meetings/travel as an advisor for Merck and Gossamer; received payment for lectures, presentations, speaker bureaus, manuscript writing, or educational events from Johnson & Johnson and Bayer; and has received research grants from Johnson & Johnson and United Therapeutics. Dr Gaine has received consulting fees for steering, advisory committee work, or speaker honoraria with AOP, Altavant, Gossamer Bio, Johnson & Johnson, Merck, and United Therapeutics. Dr Lang has relationships with drug companies including AOP Health, Johnson & Johnson, MSD, United Therapeutics, Sanofi, Medtronic, Neutrolis, and Pulnovo Medical. In addition to being an investigator in trials involving these companies, relationships include consultancy service, research grants, and membership of scientific advisory boards. Dr Chin has received consulting fees for steering, advisory or adjudication committee work with Gossamer Bio, Janssen, Merck, and United Therapeutics, and her institution has received research support for clinical studies overseen by her from Altavant, Gossamer Bio, Janssen, Merck, and United Therapeutics. Dr Pulido has received honoraria from Johnson & Johnson for consultation and lectures. His institution has received payments for clinical trials. Dr Mehta has received consulting and speaking honoraria from Gossamer Bio, Johnson & Johnson, Merck, and Pulmovant. His institution has received clinical trial support payments from Altavant, Gossamer Bio, Johnson & Johnson, and United Therapeutics. Dr Torbicki has received consulting and speaking honoraria from AOP, Bayer, Johnson & Johnson, MSD, and Pfizer; also congress participation support from AOP, Johnson & Johnson, and Pfizer. Dr McLaughlin has received grant support from Aerovate, Enzyvant, Gossamer Bio, Janssen, Merck, and SoniVie. She has served as a scientific consultant for 35Pharma, Aerami, Aerovate, Caremark, CorVista, Enzyvant, Gossamer Bio, Johnson & Johnson, Keros, Merck, Respira, United Therapeutics, and Vertex. Dr Reardon has served as a consultant and received speaking honoraria from a Johnson & Johnson company. Dr Sastry has reported that they have no relationships relevant to the contents of this paper to disclose.
