# Supplementary material for: Outcomes in Patients Receiving Treatment for Pulmonary Arterial Hypertension Associated With Repaired Congenital Heart Disease
Source: JACC Adv. 2025 Feb 24;4(3):101626. doi: 10.1016/j.jacadv.2025.101626 (PMC11908551; doi:10.1016/j.jacadv.2025.101626)
Supplement: Supplemental Material [file mmc1.docx]

# SUPPLEMENTAL APPENDIX

## SUPPLEMENTAL RESULTS

### BASELINE DEMOGRAPHIC AND CLINICAL CHARACTERISTICS

Baseline demographic and clinical characteristics of patients from GRIPHON, SERAPHIN and COMPASS-2 included in the pooled analysis cohorts are shown in **Supplemental Table 1** (Overall PAH Cohort) and **Supplemental Table 2** (CHD-PAH Cohort).

Patients’ most common congenital disorders are summarized in **Supplemental Table 3** (GRIPHON overall study population), **Supplemental Table 4** (SERAPHIN overall study population), and **Supplemental Table 5** (COMPASS-2 overall study population)

SUPPLEMENTAL TABLE 1. Demographic and Baseline Clinical Characteristics of Patients Included in the Overall PAH Cohort, Overall and According to Study Enrolled

|  | **GRIPHON  (N=1,156)** | | **SERAPHIN  (N=492)** | | **COMPASS-2 (N=334)** | | **Pooled Analysis  Overall PAH Cohort (N=1,982)** | |
| --- | --- | --- | --- | --- | --- | --- | --- | --- |
|  | **Active Drug (n=574)** | **Placebo (n=582)** | **Active Drug (n=242)** | **Placebo (n=250)** | **Active Drug (n=159)** | **Placebo (n=175)** | **Active Drug (n=975)** | **Placebo (n=1,007)** |
| Age, mean (SD) | 48 (15) | 48 (16) | 45 (15) | 47 (17) | 53 (15) | 55 (16) | 48 (15) | 49 (16) |
| Gender, n (%) |  |  |  |  |  |  |  |  |
| Male | 117 (20) | 116 (20) | 48 (20) | 65 (26) | 34 (21) | 47 (27) | 199 (20) | 228 (23) |
| Female | 457 (80) | 466 (80) | 194 (80) | 185 (74) | 125 (79) | 128 (73) | 776 (80) | 779 (77) |
| Race, n (%) |  |  |  |  |  |  |  |  |
| White | 376 (66) | 375 (64) | 135 (56) | 131 (52) | 147 (92) | 149 (85) | 658 (67) | 655 (65) |
| Non-White | 198 (34) | 207 (36) | 107 (44) | 119 (48) | 12 (7.5) | 26 (15) | 317 (33) | 352 (35) |
| BMI, mean kg/m^2^ (SD) | 26.9 (6.4) | 26.7 (6.1) | 25.6 (6.1) | 25.2 (5.1) | 28.5 (6.8) | 29.1 (6.9) | 26.8 (6.4) | 26.7 (6.2) |
| Mean time from PAH diagnosis, months (SD) | 28 (42) | 30 (45) | 31 (44) | 31 (45) | 25 (47) | 26 (51) | 28 (43) | 29 (46) |
| WHO FC, n (%) |  |  |  |  |  |  |  |  |
| I | 4 (0.7) | 5 (0.9) | 1 (0.4) | 0 (0) | 0 (0) | 0 (0) | 5 (0.5) | 5 (0.5) |
| II | 274 (48) | 255 (44) | 120 (50) | 130 (52) | 71 (45) | 69 (39) | 465 (48) | 454 (45) |
| III | 293 (51) | 314 (54) | 116 (48) | 116 (46) | 88 (55) | 104 (59) | 497 (51) | 534 (53) |
| IV | 3 (0.5) | 8 (1.4) | 5 (2.1) | 4 (1.6) | 0 (0) | 2 (1.1) | 8 (0.8) | 14 (1.4) |
| Concomitant PAH therapy, n (%) |  |  |  |  |  |  |  |  |
| Missing | 5 (0.9) | 0 | 0 | 0 | 0 | 0 | 5 (0.5) | 0 |
| No | 107 (19) | 124 (21) | 88 (36) | 96 (38) | 0 | 0 | 195 (20) | 220 (22) |
| Yes | 462 (80) | 458 (79) | 154 (64) | 154 (62) | 159 (100) | 175 (100) | 775 (79) | 787 (78.2) |

BMI = body mass index; FC = functional class; PAH = pulmonary arterial hypertension; SD = standard deviation; WHO = World Health Organization.

SUPPLEMENTAL TABLE 2. Demographic and Baseline Clinical Characteristics of Patients Included in the repaired CHD-PAH Cohort, Overall and According to Study Enrolled

|  | **GRIPHON  (N=110)** | | **SERAPHIN  (N=47)** | | **COMPASS-2  (N=20)** | | **Pooled Analysis  CHD-PAH Cohort (N=177)** | |
| --- | --- | --- | --- | --- | --- | --- | --- | --- |
|  | **Active drug (n=60)** | **Placebo (n=50)** | **Active drug (n=21)** | **Placebo (n=26)** | **Active drug (n=9)** | **Placebo (n=11)** | **Active drug (n=90)** | **Placebo**  **(n=87)** |
| Age, mean (SD) | 40 (15) | 40 (15) | 42 (18) | 35 (15) | 40 (13) | 46 (12) | 41 (16) | 39 (15) |
| Gender, n (%) |  |  |  |  |  |  |  |  |
| Male | 14 (23) | 8 (16) | 4 (19) | 12 (46) | 2 (22) | 1 (9) | 20 (22) | 21 (24) |
| Female | 46 (77) | 42 (84) | 17 (81) | 14 (54) | 7 (78) | 10 (91) | 70 (78) | 66 (76) |
| Race, n (%) |  |  |  |  |  |  |  |  |
| White | 35 (58) | 29 (58) | 11 (52) | 16 (62) | 9 (100) | 8 (73) | 55 (61) | 53 (61) |
| Non-White | 25 (42) | 21 (42) | 10 (48) | 10 (38) | 0 (0) | 3 (27) | 35 (39) | 34 (39) |
| BMI, mean kg/m^2^ (SD) | 25.8 (6.7) | 23.9 (5.5) | 23.9 (5.0) | 24.1 (3.9) | 27.9 (6.5) | 26.7 (5.6) | 25.6 (6.4) | 24.3 (5.1) |
| Mean time from PAH diagnosis, months (SD) | 43 (73) | 42 (66) | 46 (36) | 75 (97) | 90 (165) | 75 (100) | 49 (81) | 56 (82) |
| WHO FC, n (%) |  |  |  |  |  |  |  |  |
| I | 1 (1.7) | 0 (0) | 0 (0) | 0 (0) | 0 (0) | 0 (0) | 1 (1) | 0 (0) |
| II | 38 (63) | 28 (56) | 12 (57) | 15 (58) | 4 (44) | 7 (64) | 54 (60) | 50 (57) |
| III | 21 (35) | 22 (44) | 9 (43) | 11 (42) | 5 (56) | 4 (36) | 35 (39) | 37 (43) |
| IV | 0 | 0 | 0 | 0 | 0 | 0 | 0 | 0 |
| Concomitant PAH therapy, n (%) |  |  |  |  |  |  |  |  |
| Missing | 0 | 0 | 0 | 0 | 0 | 0 | 0 | 0 |
| No | 20 (33) | 15 (30) | 9 (43) | 13 (50) | 0 | 0 | 29 (32) | 28 (32) |
| Yes | 40 (67) | 35 (70) | 12 (57) | 13 (50) | 9 (100) | 11 (100) | 61 (68) | 59 (68) |

BMI = body mass index; CHD = congenital heart defect; FC = functional class; PAH = pulmonary arterial hypertension; SD = standard deviation; WHO = World Health Organization.

SUPPLEMENTAL TABLE 3. Patients’ Most Common Congenital Disorders Recorded at Baseline in GRIPHON (occurring in ≥2 subjects overall)

| **System Organ Class/Preferred Term**  **n (%) of patients** | **Active Drug  (n=574)** | **Placebo  (n=582)** |
| --- | --- | --- |
| **Congenital, familial, and genetic disorders** | **75 (13.1)** | **64 (11.0)** |
| Atrial septal defect | 27 (4.7) | 21 (3.6) |
| Heart disease congenital (other) | 10 (1.7) | 6 (1.0) |
| Patent ductus arteriosus | 6 (1.0) | 2 (0.3) |
| Anomalous pulmonary venous connection | 5 (0.9) | 2 (0.3) |
| Ventricular septal defect | 4 (0.7) | 8 (1.4) |
| Eisenmenger’s syndrome | 4 (0.7) | 1 (0.2) |
| Hereditary hemorrhagic telangiectasia | 2 (0.3) | 4 (0.7) |
| Kidney duplex | 2 (0.3) | 1 (0.2) |
| Pectus excavatum | 2 (0.3) | 1 (0.2) |
| Gilbert’s syndrome | 1 (0.2) | 3 (0.5) |
| Methylenetetrahydrofolate reductase deficiency | 1 (0.2) | 2 (0.3) |
| Bicuspid aortic valve | 1 (0.2) | 1 (0.2) |
| Congenital hypercoagulation | 1 (0.2) | 1 (0.2) |
| Cryptorchism | 1 (0.2) | 1 (0.2) |
| Factor V Leiden mutation | 1 (0.2) | 1 (0.2) |
| Gastrointestinal arteriovenous malformation | 1 (0.2) | 1 (0.2) |
| Malformation venous | 1 (0.2) | 1 (0.2) |
| Protein S deficiency | 1 (0.2) | 1 (0.2) |
| Thalassemia | 1 (0.2) | 1 (0.2) |
| Renal aplasia | 0 | 2 (0.3) |
| Renal hypoplasia | 0 | 2 (0.3) |

SUPPLEMENTAL TABLE 4. Patients’ Most Common Congenital Disorders Recorded at Baseline in SERAPHIN (occurring in ≥2 subjects overall)

| **System Organ Class/Preferred Term**  **n (%) of patients** | **Active Drug^a^ (n=242)** | **Placebo  (n=250)** |
| --- | --- | --- |
| **Congenital, familial, and genetic disorders** | **21 (8.7)** | **23 (9.2)** |
| Atrial septal defect | 11 (4.5) | 9 (3.6) |
| Ventricular septal defect | 2 (0.8) | 7 (2.8) |
| Patent ductus arteriosus | 1 (0.4) | 2 (0.8) |
| Heart disease congenital (other) | 1 (0.4) | 1 (0.4) |

^a^Only patients receiving macitentan at the 10-mg dose were included in the active treatment group.

SUPPLEMENTAL TABLE 5. Patients’ Most Common Congenital Disorders Recorded at Baseline in COMPASS-2 (occurring in ≥1 subjects overall)

| **System Organ Class/Preferred Term**  **n (%) of patients** | **Active drug (N = 159)** | **Placebo  (N = 175)** |
| --- | --- | --- |
| **Congenital, familial, and genetic disorders** | **13 (8.2)** | **16 (9.1)** |
| Atrial septal defect | 7 (4.4) | 7 (4.0) |
| Atrioventricular septal defect | 1 (0.6) | 0 |
| Congenital hip deformity | 1 (0.6) | 0 |
| Deafness congenital | 1 (0.6) | 0 |
| DiGeorge syndrome | 1 (0.6) | 0 |
| Hip dysplasia | 1 (0.6) | 0 |
| Methylenetetrahydrofolate reductase deficiency | 1 (0.6) | 0 |
| Renal aplasia | 1 (0.6) | 0 |
| Truncus arteriosus persistent | 1 (0.6) | 0 |
| Heart disease congenital (other) | 0 | 2 (1.1) |
| Arteriovenous malformation | 0 | 1 (0.6) |
| Coarctation of the aorta | 0 | 1 (0.6) |
| Congenital hypercoagulation | 0 | 1 (0.6) |
| Factor V Leiden mutation | 0 | 1 (0.6) |
| Hypertrophic cardiomyopathy | 0 | 1 (0.6) |
| Neurofibromatosis | 0 | 1 (0.6) |
| Patent ductus arteriosus | 0 | 1 (0.6) |
| Talipes | 0 | 1 (0.6) |
| Ventricular septal defect | 0 | 1 (0.6) |
